# A Unique Presentation of Gingival Melanoacanthoma: Case Report and Review of Literature

**DOI:** 10.7759/cureus.7315

**Published:** 2020-03-18

**Authors:** Archita Datta, Arundeep K Lamba, Shruti Tandon, Aadithya Urs, Mahima LNU

**Affiliations:** 1 Periodontics, Maulana Azad Institute of Dental Sciences, New Delhi, IND; 2 Oral Pathology and Microbiology, Maulana Azad Institute of Dental Sciences, New Delhi, IND

**Keywords:** gingiva, pigmentation, laser, immunohistochemistry, melanoacanthoma

## Abstract

Melanoacanthoma is a benign, deeply pigmented condition of the oral mucosa characterized by the proliferation of melanocytes and keratinocytes. A 60-year-old male, with no history of systemic diseases or medical treatment, presented with an asymptomatic diffuse pigmentation involving the maxillary and mandibular gingiva. The histopathology of the anomalous pigmented area was analyzed and the dendritic melanocytes expressed positively for Masson-Fontana, S-100, and HMB-45. The clinical and microscopic findings were indicative of melanoacanthoma. Depigmentation with a diode laser was performed in the areas of esthetic concern. A regular periodic screening was done to rule out any alteration in color, size, and shape. A one-year follow-up disclosed no new lesions. The observations noted in this case are rare, and our literature review identified only a single previously documented case of gingival melanoacanthoma in the Indian subpopulation.

## Introduction

Melanoacanthoma is an uncommon, reactive, pigmented mucocutaneous lesion characterized by the presence of keratinocytes admixed with dendritic melanocytes. An alarming feature of oral melanoacanthoma is its rapid growth rate, potentially masquerading as a melanoma. The first evidence of cutaneous melanoacanthoma was described by Bloch in 1927 [1]. Mishina and Pinkus coined the term melanoacanthoma in 1960 and observed that this lesion could occur in both the skin and the mucosa [1]. The etiology is uncertain, and it may represent a physiologic or reactive process.

The buccal mucosa is the most commonly reported intraoral site of melanoacanthoma (51.4%), with fewer lesions originating on the palate (22.2%), lips (15.2%), and gingiva (5.6%) [2]. Oral lesions clinically represent as smooth macules or slightly raised papules with possible color variations ranging from brown to blue-black. Oral melanoacanthoma is usually seen in blacks, with a female predilection and is most common between the third and fourth decades of life [3]. Herein, we discuss an unusual case report of gingival melanoacanthoma in an Indian male, which presented clinically as multifocal pigmented lesions.

## Case presentation

**Figure 1:**
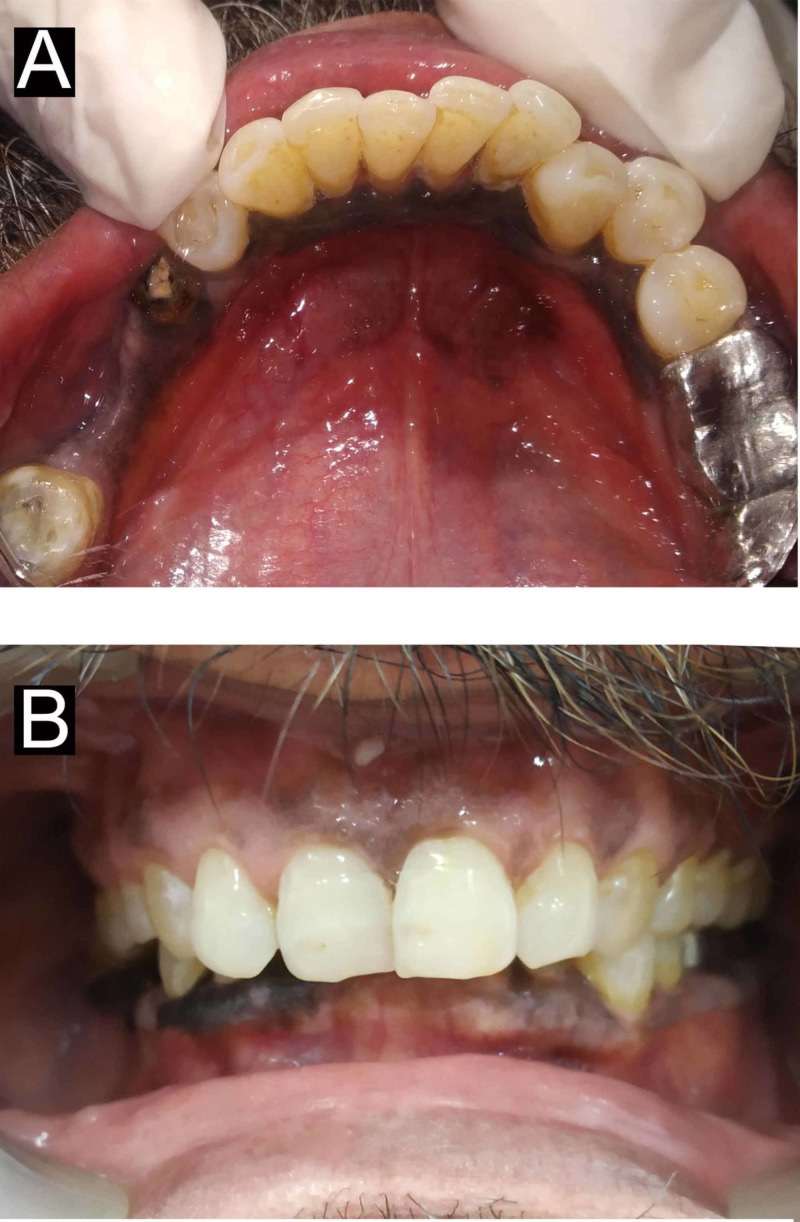
Preoperative view (A) Preoperative lingual view showing pigmentation extending from the lower left to the lower right molar; (B) Preoperative frontal view showing pigmentation in the lower anterior region

A sixty-year-old male patient reported to the department of periodontics Maulana Azad Institute of Dental Sciences (MAIDS) with a chief complaint of pigmentation of the gums in the lower arch. The patient first noted the pigmentation due to the dislodgement of his prosthesis in the 46 region one month ago. He was unaware of the onset of the pigmentation, however, the extent and appearance made the patient anxious. Subsequently, the patient reported to the hospital to negate any grievous condition. Extraoral examination revealed no relevant findings. Intraoral examination revealed multiple, widespread gingival macular bluish-black pigmentations on the buccal surface of 42-47 and lingually from 37-47. The edentulous region also showed a diffuse area of pigmentation (Figure 1).

**Figure 2:**
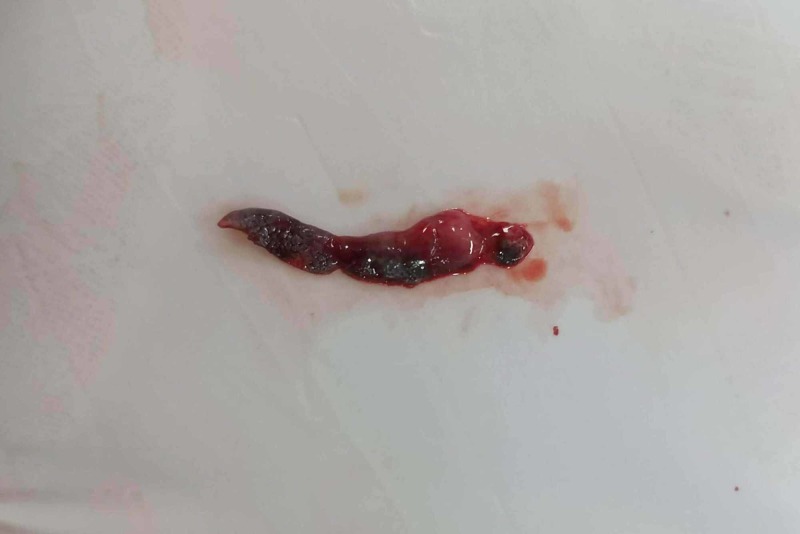
Gross biopsied specimen

The pigmented area was smooth, non-tender with irregular margins involving free and attached gingiva. The patient denied any cutaneous pigmentary changes, foods, or dental hygiene agents that had caused any oral irritation, usage of tobacco products, and awareness of oral habits of compulsion. The widespread nature and clinical appearance of the lesion were perplexing for the authors, thereby necessitating an incisional biopsy. The area to be biopsied was the edentulous region (46). The gross specimen comprised an irregular, blackish-brown soft tissue measuring 1.5 x 0.6 x 0.2 cm in size (Figure 2).

**Figure 3:**
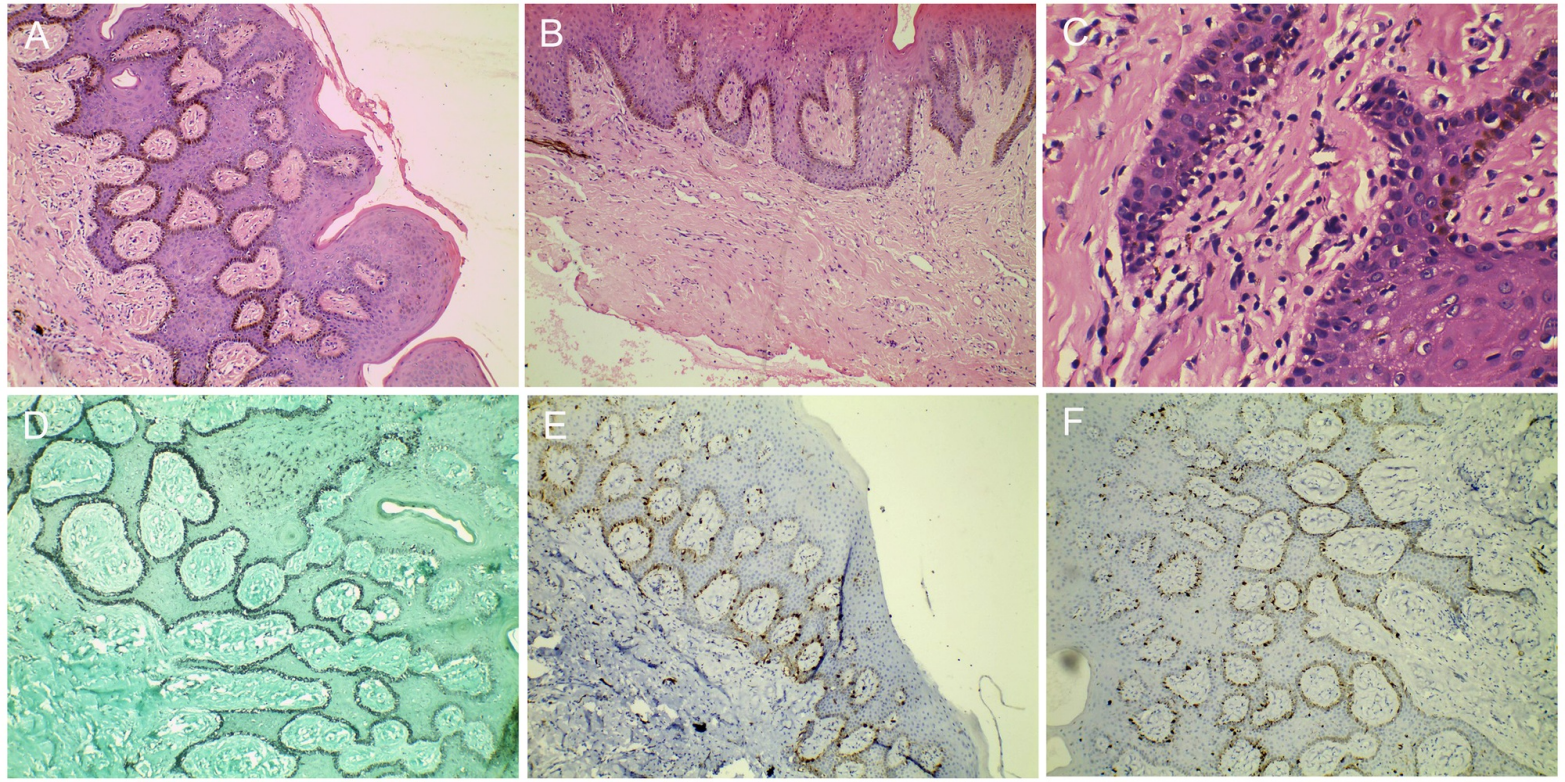
Photomicrographs of immunohistochemistry and hematoxylin and eosin staining (A) Photomicrograph of melanoacanthoma at low magnification (40X) exhibiting numerous melanocytes with melanosomes throughout the entire basal extension; (B) Photomicrograph, at low magnification (40X) depicting increased number of melanocytes in the basal layer along with melanin incontinence; (C) Photomicrograph, at higher magnification (100X) showing the presence of numerous dendritic cells that contain brownish pigments in their cytoplasm, dispersed throughout the entire extension of the epithelial tissue; (D) Photomicrograph, at low magnification showing melanocytes expressing positivity for Masson-Fontana in the basal and parabasal cell layer; (E) Photomicrograph, at low magnification showing S-100 positive melanocytes in the basal and parabasal cell layer; (F) Photomicrograph, at low magnification showing S-100 and HMB45 positive melanocytes in the basal and parabasal cell layer

On microscopic examination, the studied section showed predominantly hyperplastic, hyperorthokeratinized, stratified squamous epithelium exhibiting long and narrow rete ridges. Spongiosis and mild acanthosis were also noted. Numerous benign dendritic melanocytes were scattered throughout the lesional epithelium, chiefly within the basal cell layers. A few dendritic melanocytes were also present within the stratum spinosum. Abundant melanin pigmentation within the basal keratinocytes was noted. Many areas of melanin incontinence were also observed within the juxtaepithelial and superficial connective tissue (Figures 3A-3C).

The dendritic melanocytes expressed positively for Masson-Fontana, S-100, and HMB-45 (Figures 3D-3F). The supporting moderately fibrocollagenous connective tissue comprised an irregular arrangement of collagen fibers, many fibroblasts, and many blood vessels of varying sizes. The microscopic findings of the incisional biopsy were suggestive of melanoacanthoma.

After establishing the diagnosis, the patient was reassured of the non-malignant nature of the lesion and was advised regular follow-ups.

**Figure 4:**
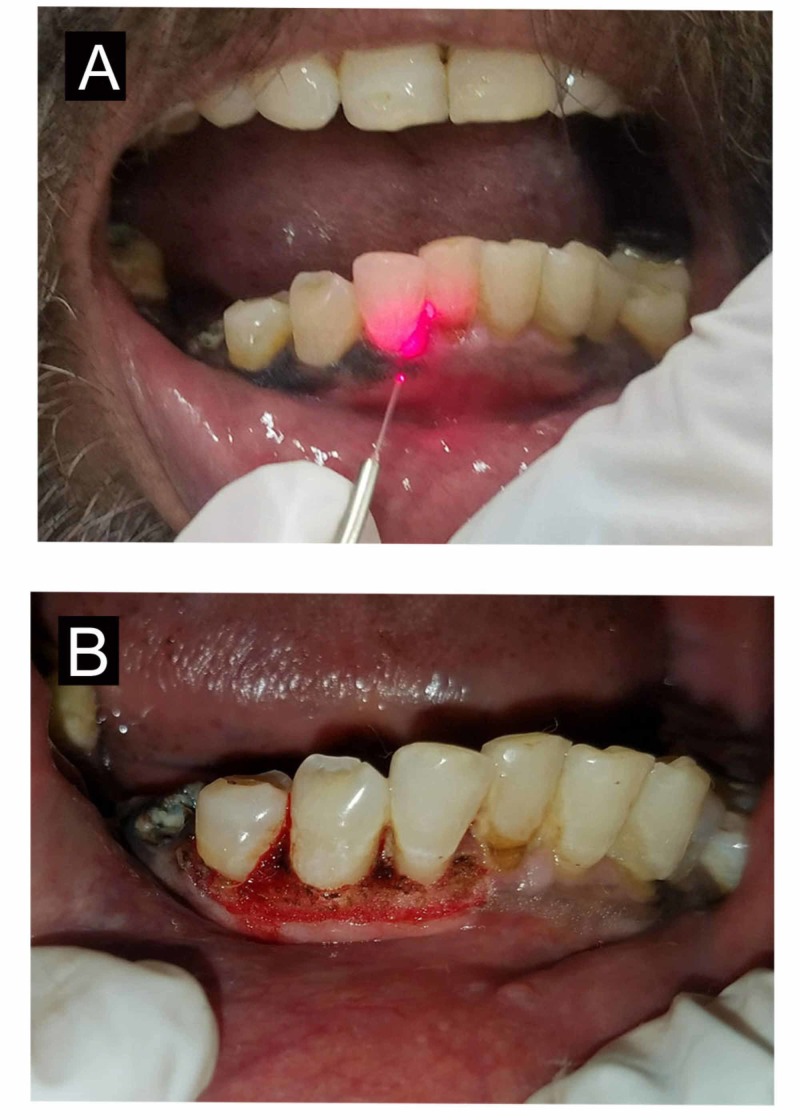
Depigmentation with diode laser (A) Depigmentation using diode laser; (B) immediate postoperative view

To address the patient’s esthetic concerns, depigmentation was performed in the 42-45 region using a diode laser (Picasso™; AMD Lasers; West Jordan, UT) under local anesthesia (1:80000) (Figure 4).

**Figure 5:**
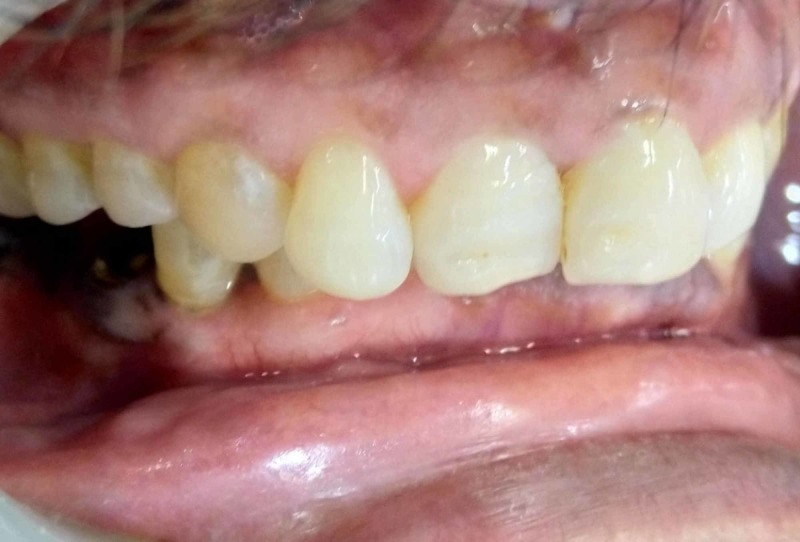
Postoperative view One-year follow-up with no recurrence

Postoperative evaluation was done after seven days and healing was uneventful. After one year of follow-up, there was no significant recurrence (Figure 5).

## Discussion

Inclusive of the present case, 16 patients with gingival melanoacanthomas were documented. In order to review the literature, a summary of prior reported cases were tabulated (Table 1).

**Table 1:** Reported cases of gingival melanoacanthoma

Authors	Year	Age/Gender	Race or ethnicity	Clinical presentation	Site of lesion	Outcome at follow-up
Goode et al. [4]	1983	36/F	African American	Brown lesion	Attached gingiva	Resolution
Maize et al. [5]	1988	NA/F	African American	Pigmented macules	Mandibular gingiva	NA
Flaitz et al. [6]	2000	40/F	African American	Brownish black	Mandibular gingiva	NA
Fornatora et al. [7]	2003	72/F	Caucasian	NA	Mandibular gingiva	Recurrence
Carlos‑Bregni et al. [8]	2007	M/7	Caucasian	Pigmented macule	Mandibular gingiva	Resolution
Carlos‑Bregni et al. [8]	2007	33/F	Hispanic	Dark Brown macule	Maxillary gingiva	NA
Yarom; Hirshberg; Buchner [2]	2007	60/ F	Caucasian	Brown macule	Maxillary gingiva	Resolution
Najjar and Chiodo [9]	2008	39/M	NA	NA	Mandibular gingiva	NA
Brooks and Nikitakis [10]	2008	47/M	African American	Pigmented macule	Maxillary gingiva	NA
Brooks et al. [11]	2009	NA/F	Caucasian	Brown macule	Maxillary and mandibular gingiva	Recurrence
Marocchio et al. [12]	2009	74/ F	African American	Black/ Brownish	Buccal mucosa, lips, gingiva, and tongue	Resolution
Tapia et al. [13]	2011	60/F	Hispanic	Brown macule	Maxillary gingiva	Resolution
Kennedy Babu et al. [14]	2013	13/M	Asian	Brownish black	Maxillary and mandibular gingiva	Resolution
Dantas et al. [15]	2017	50/F	African American	Brown and black	Gingiva, tongue, and buccal mucosa	Resolution
Gonçalves et al. [16]	2019	53/F	Caucasian	Brownish black	Gingiva and upper lip	Recurrence
Present case	2019	60/M	Asian	black	gingiva	Resolution

It occurs mainly in Blacks, followed by Caucasians, Hispanics, and, rarely, in Asians. In contrast to the female gender predilection (75%), the case presented here is that of an Indian male. Based on the cumulative data, the majority of patients present with a solitary lesion (13 out of 16) whereas three cases noted multiple lesions. To the best of our knowledge, this is the first reported case of multifocal gingival melanoacanthoma in an adult Indian male. The prognosis of oral melanoacanthoma is excellent, however, in three of the reviewed cases, the authors reported recurrence [7,12]. No evidence of recurrence and no alteration in existing lesions were observed after the one-year follow-up.

The definitive pathogenesis of melanoacanthoma has not been identified, although these lesions are generally considered to be reactive in origin [17]. The etiology has been attributed to chronic local irritation or mild trauma. Most oral melanoacanthomas occur on stress-bearing surfaces (e.g., the palate or alveolar ridge) or common sites of trauma such as the buccal mucosa [18]. In the present case, the presence of a fixed prosthesis could have been a source of chronic irritation to the adjacent tissues, resulting in diffused pigmentation.

Melanoma, post-inflammatory lichen planus, oral melanotic macule, acquired melanocytic nevus, hemochromatosis, and so on have a similar clinical appearance to melanoacanthoma and are crucial for the differential diagnosis. Other conditions associated with increased melanin deposition include physiologic racial pigmentation, Addison disease, and syndromes such as McCune-Albright and Peutz-Jeghers ​​​​[19]. Consumption of various heavy metals and the administration of amiodarone, quinine derivatives, estrogens, phenothiazines, minocycline, ketoconazole, and zidovudine can present as a pigmented lesion of the oral cavity. Exogenous sources causing the discoloration of the gingiva include amalgam and graphite implantation, khat chewing, tattooing, and various oral hygiene products.

All of the biopsied specimens seen with our case were consistent with the histopathologic features of melanoacanthoma. Immunostaining for HMB-45 and S-100 highlighted the presence of benign melanocytes and dendritic melanocytes and was in agreement with previous investigations. Dendritic melanocytes are not pathognomonic for melanoacanthoma, however, they may occur with various neoplasms, most notably melanoma, squamous cell carcinoma, mucoepidermoid carcinoma, blue nevus, and Spitz nevus [20].

Based on the features described in the available literature, oral melanoacanthoma (OMA) is a rare, reactive lesion affecting the oral mucous membrane with no malignant potential. Its treatment should be directed towards the elimination of all local potentially irritating factors and excluding any other cause of oral pigmentation.

## Conclusions

Taking everything into account, gingival melanoacanthoma is an uncommonly encountered pigmented lesion and should undergo a histopathologic examination for timely identification to rule out malignancy. This case report underlines the relevance for clinicians to consider oral melanoacanthoma in the differential diagnosis of multifocal gingival pigmentations.
